# A Case of Kikuchi-Fujimoto Disease Associated with Erosive Lichen Planus

**DOI:** 10.7759/cureus.7312

**Published:** 2020-03-18

**Authors:** Shoroq Alosaimi, Bashayr Hijazi, Ahmed Alhumidi, Fahad Alsaif

**Affiliations:** 1 Department of Dermatology, College of Medicine, Majmaah University, Majmaah, SAU; 2 College of Medicine, Alfaisal University, Riyadh, SAU; 3 Department of Pathology, College of Medicine, King Saud University, Riyadh, SAU; 4 Department of Dermatology, College of Medicine, King Saud University, Riyadh, SAU

**Keywords:** histiocytic necrotizing lymphadenitis, kikuchi disease, lichen planus

## Abstract

Kikuchi-Fujimoto disease (KFD), also known as histiocytic necrotizing lymphadenitis, is a benign, self-limiting disorder with unknown etiology. The most frequent clinical manifestations include lymphadenopathy, fever, cutaneous lesions, arthritis, fatigue, and hepatosplenomegaly. Cutaneous manifestations of KFD, occurring in about one-third of patients, are typically non-specific, rarely presenting as symmetrically distributed lesions. The prevalence of erosive lichen planus in patients with KFD, as of this date, is unknown with no previously reported cases describing an association between the two conditions. In the following case report, we report a case presenting with bilateral symmetrical erosive lichen planus of the heel associated with KFD as being a possible, rather novel, cutaneous manifestation.

## Introduction

KFD, also known as histiocytic necrotizing lymphadenitis or Kikuchi-Fujimoto disease, was introduced by Kikuchi and Fujimoto independently in 1972 [1]. It is a rare, benign, self-limiting condition with a good prognosis [2]. Although reported worldwide in a variety of ethnic groups, KFD classically affects young women with the highest prevalence being in Asia [3-5]. 

The most common clinical features include cervical lymphadenopathy, fever, and cutaneous eruptions with or without systemic symptoms [6]. The most common cutaneous manifestations of KFD are nonspecific skin rashes, erythematous maculopapular lesions, nodules, erythematous papules, indurated erythematous lesions, and erythema multiforme with areas on the face and upper trunk most frequently affected [2, 5].

The presentation of KFD can mimic a number of serious conditions, including lymphoma, tuberculosis, human immunodeficiency virus (HIV), toxoplasmosis, and systemic lupus erythematosus (SLE), with this overlapping similarity making it a diagnostic challenge [7]. Herein, we present a case of a young male with bilateral symmetrical erosive lichen planus of the heel associated with KFD.

## Case presentation

An 18-year-old Saudi male was referred to the Department of Dermatology at King Khalid University Hospital in 2017 with a six-year history of bilateral symmetrical painful heel ulceration. Prior to his presentation, the patient had been following up at several hospitals wherein no definitive diagnosis had been reached and he had been given numerous treatment regimens with no adequate response. Past medical, surgical, and medication histories were unremarkable. Family history was positive for parental consanguinity, SLE, and thyroid disease. He was a non-smoker with no high-risk behavior. 

**Figure 1:**
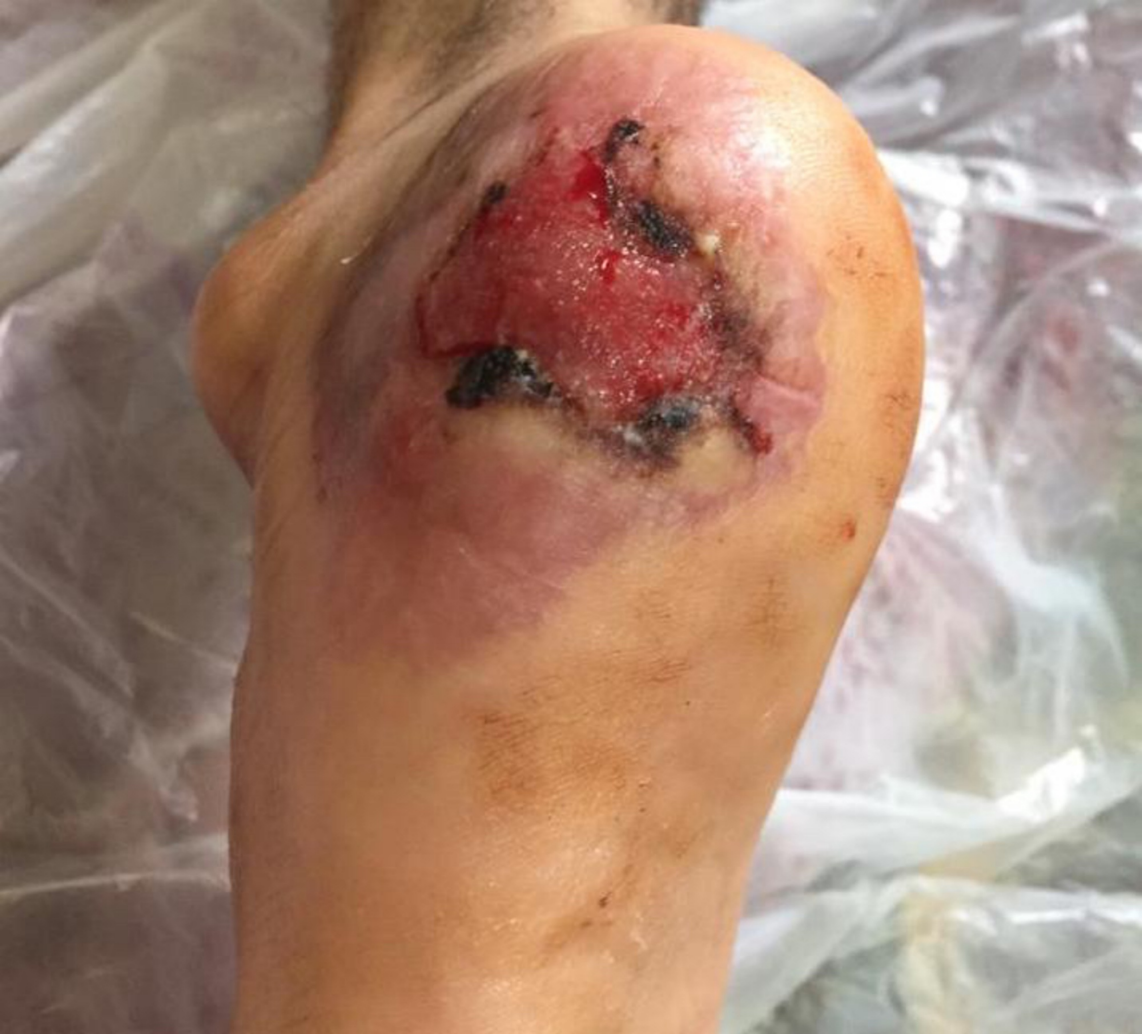
Tender heel ulcer with a central eschar and a peripheral rim of violet erythema about 5 x 4 cm in diameter

Physical examination revealed bilateral, symmetrical, tender heel ulcers with central eschar and a peripheral rim of violet erythema about 5 x 4 cm in diameter (Figure 1). Differential diagnoses included pyoderma gangrenosum, vasculitis, and erosive lichen planus.

**Figure 2:**
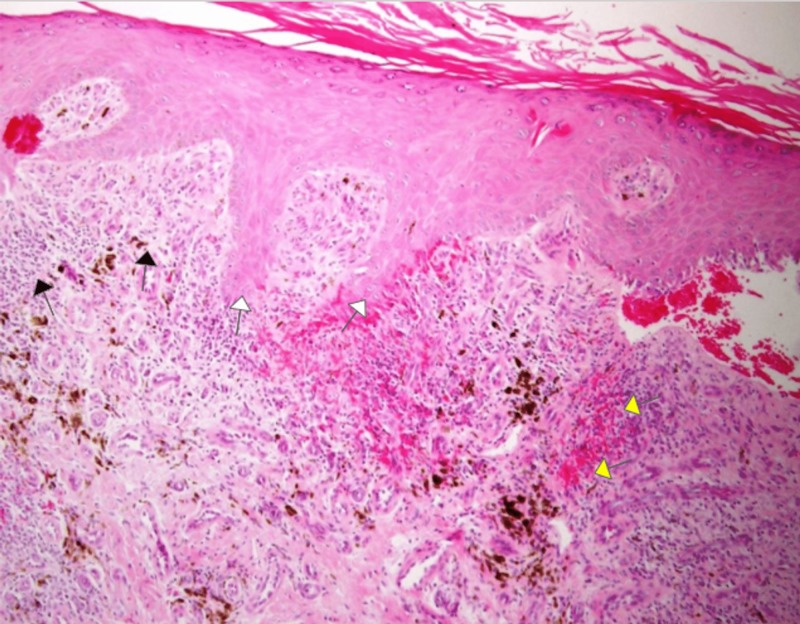
Hematoxylin and eosin (H&E) stain from biopsy Skin punch biopsy showing band-like lymphohistiocytic infiltrate (black arrows) at the dermoepidermal junction associated with saw tooth rete ridges (white arrows), hypergranulosis, and orthokeratosis. Extravasated red blood cells (RBCs) are also noted (yellow arrows). (H&E stain, original magnification x 100).

Upon investigation, multiple bacterial cultures taken from the heel ulcer showed no evidence of bacterial infection, and a skin biopsy showed features suggestive of erosive lichen planus (Figure 2).

**Figure 3:**
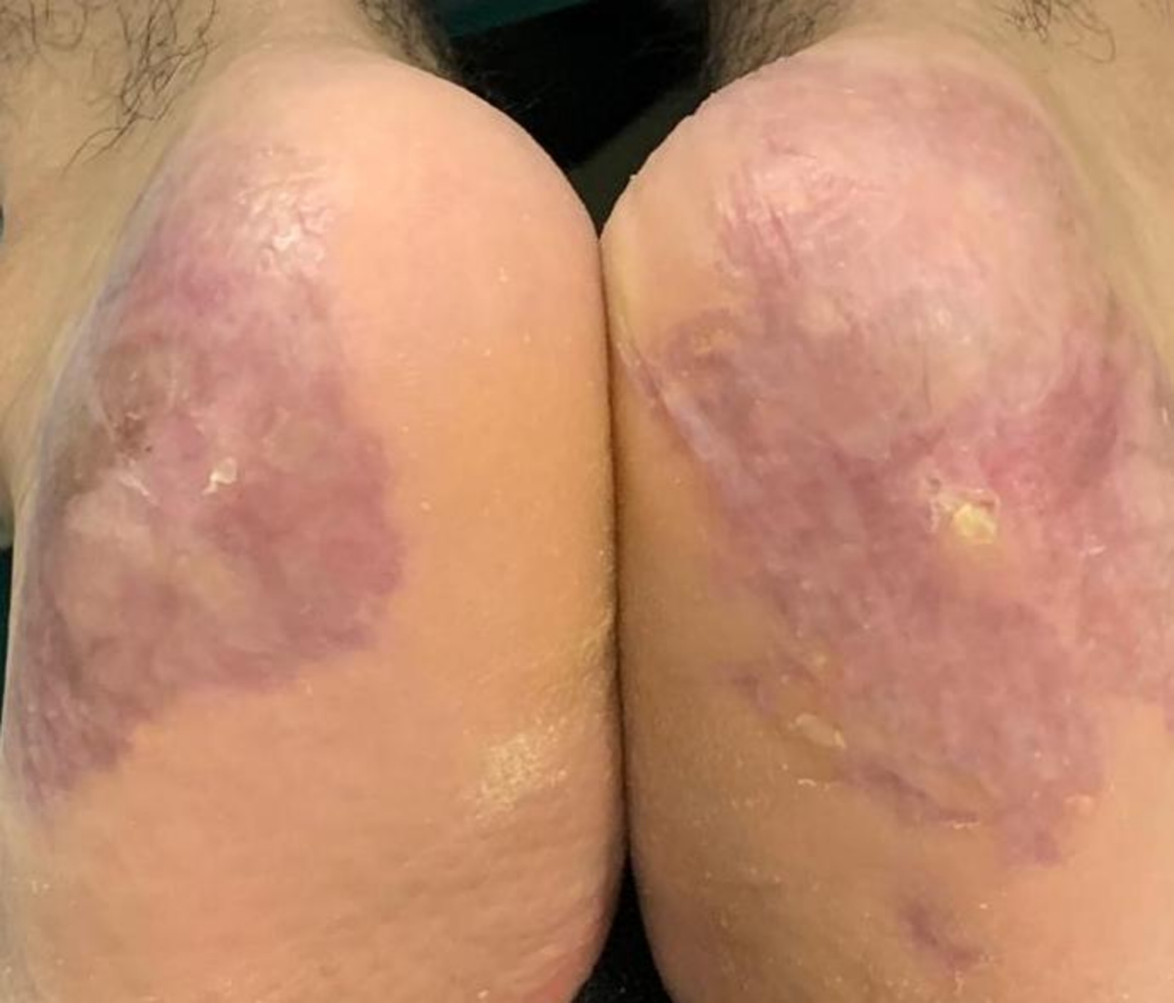
Bilateral heel ulcerations healed with atrophic scarring

The initial treatment regimen included appropriate wound care and systemic prednisolone, 0.5 mg/kg, for two months with only partial improvement. Treatment was then changed to cyclosporine, 3 mg/kg, for six months after which the heel ulcerations healed with atrophic scarring (Figure 3). Multiple relapses occurred throughout the course of the following two years with variable response to prednisolone and cyclosporine.

**Figure 4:**
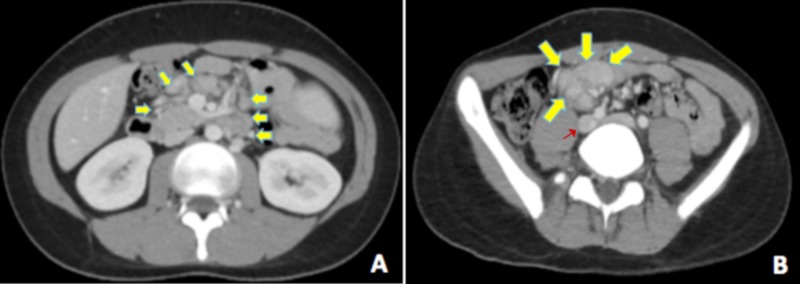
Computed tomography (CT) scan of the abdomen A CT scan in the axial plane showed multiple, enlarged mesenteric adenopathies in a pattern of rounded nodular enhancing masses (yellow arrows) seen at the mesenteric root (4A) and along the right iliac vessels (4B, red arrow).

After two to three years of follow-up for the aforementioned cutaneous manifestations, the patient developed a new constellation of symptoms, including unexplained weight loss, high-grade fever, night sweats, generalized skin rash, generalized intermittent abdominal pain, and diarrhea. Computed tomography (CT) scan of the abdomen showed multiple intra-abdominal lymphadenopathy, after which the patient was admitted, given symptomatic treatment (paracetamol, 1 gm every 8 hrs), and underwent a thorough investigation (Figure 4). Differential diagnoses at that point included tuberculosis, lymphoma, connective tissue disease, and inflammatory bowel disease.

Laboratory investigations showed normal complete blood count (CBC) with differential count and liver function tests. He had high erythrocyte sedimentation rate (ESR) - 66, high antinuclear antibody (ANA) (1:2,560) with coarse speckled pattern, high rheumatoid factor - 25, and a high anti-ribonucleoprotein (RNP) antibody. Microbiological workup for tuberculosis, brucellosis, typhoid fever, hepatitis B and C, and HIV came back negative. Brain magnetic resonance imaging (MRI) was normal. Upper and lower gastrointestinal (GI) endoscopy were normal. A terminal ileum biopsy showed unremarkable ileal mucosa. 

**Figure 5:**
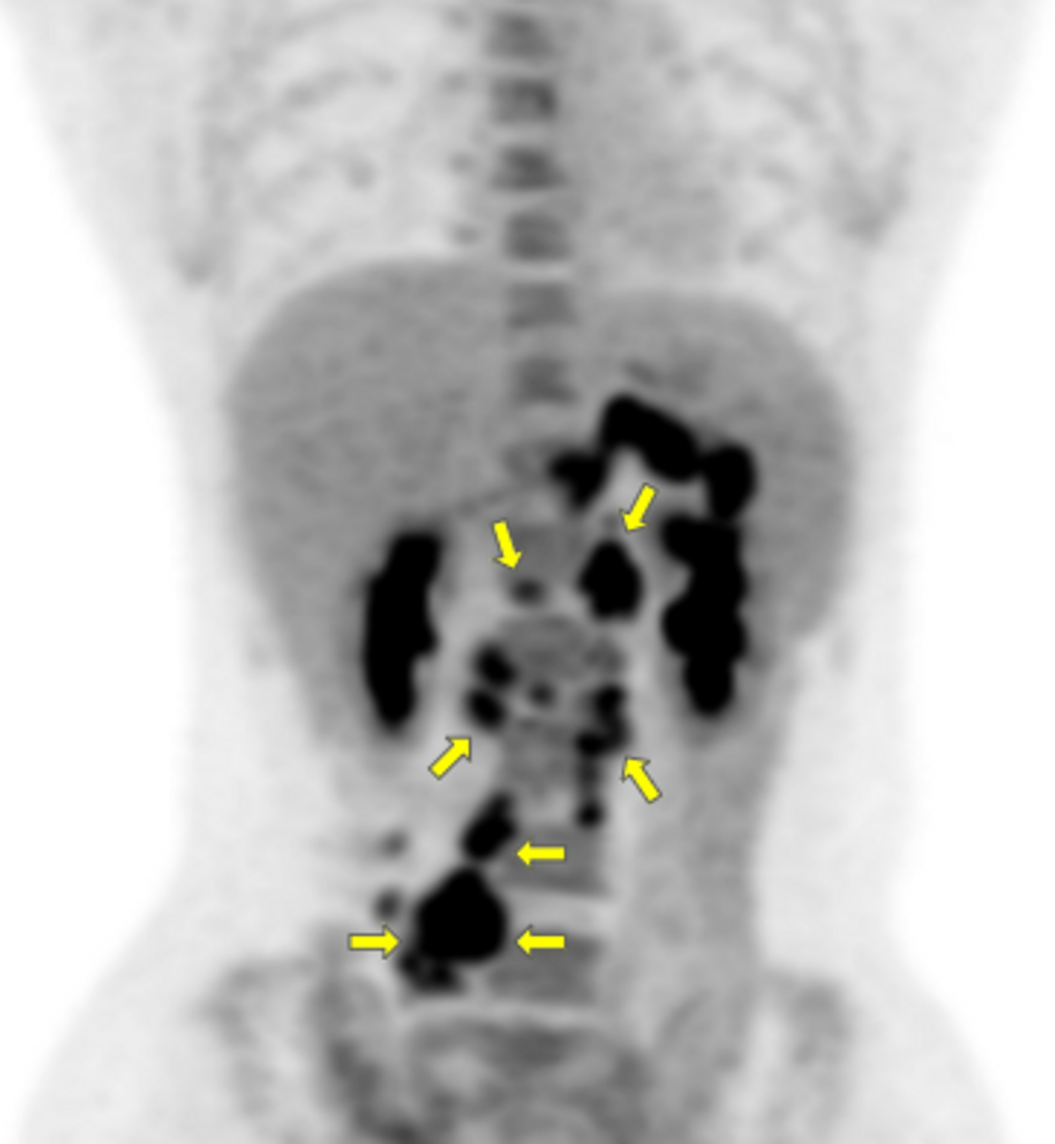
Positron emission tomography (PET) scan with maximum intensity projection (MIP) PET with MIP shows extensive sites of involvement visualized as areas of fluorodeoxyglucose (FDG) uptake in the upper abdomen and mesenteric and retroperitoneal lymph nodes (yellow arrows)

After hematology/oncology consultation, positron emission tomography (PET) scan and excisional lymph node biopsy were done to rule out lymphoma. A PET scan showed hypermetabolic lymphadenopathy below the diaphragm, in keeping with lymphoma (Figure 5).

**Figure 6:**
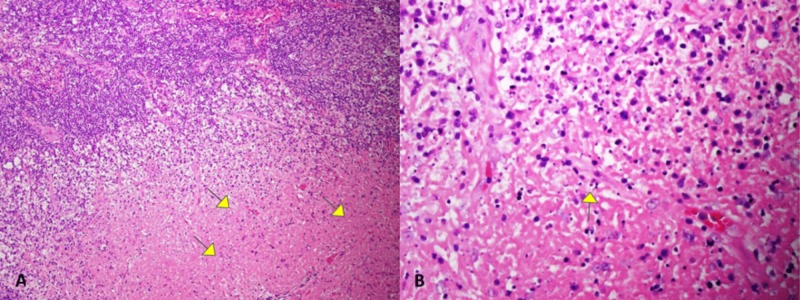
Excisional lymph node biopsy A) Focal necrosis (yellow arrows), karyorrhexis, and fibrin material (hematoxylin and eosin (H&E) stain, original magnification x 100); B) higher power view of karyorrhexis (yellow arrow) and lymph node necrosis (H&E stain, original magnification x 400)

Excisional abdominal lymph node biopsy showed necrotizing lymphadenitis with focal areas of necrosis and karyorrhexis with a differential diagnosis of KFD and connective tissue diseases, including SLE (Figure 6).

**Figure 7:**
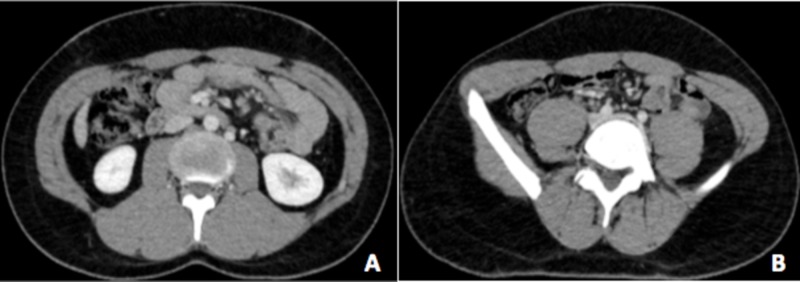
Follow-up computed tomography (CT) scan of the abdomen six months post-discharge showing significant regression of abdominal lymphadenopathy

Upon further rheumatologic evaluation, overall features were more suggestive of KFD rather than SLE with a potential of overlap between the two conditions. After discharge, the patient recovered well off the medication with spontaneous resolution. A repeat CT scan of the abdomen six months post-discharge showed an interval regression of the abdominal lymph nodes (Figure 7).

## Discussion

KFD, also known as histiocytic necrotizing lymphadenitis, is a rare, benign, idiopathic, and generally self-limited cause of lymphadenitis that is characterized by subacute necrotizing regional lymphadenopathy. Although initially described in Japan in 1972, KFD has since been reported worldwide in a variety of ethnic backgrounds [1]. It usually occurs in adults below the age of 40, although it can affect any age group. Both genders can be affected, with most reports showing a female predominance [2, 5, 8].

The clinical course is acute to subacute with the most common clinical features, including cervical lymphadenopathy, fever, and cutaneous eruptions.

The most common region of lymph node involvement is posterior cervical (60% - 90% of cases) and frequently occurs with concomitant involvement of axillary and/or supraclavicular lymph nodes. Generalized lymphadenopathy, however, is rarely reported [2, 5, 8-9]. Intra-abdominal regions have also been reported with unknown frequency. Isolated intra-abdominal lymphadenopathy, as presented in our case, was reported in only 2.6% - 4.3% of cases [2, 10]. Lymphadenopathy is most frequently associated with fever and other less commonly reported signs and symptoms, including weight loss, vomiting, headache, arthralgia, night sweats, sore throat, hepatosplenomegaly, and cutaneous lesions [2, 8-9]. Cutaneous eruptions in KFD occur in about 40% of cases with reported manifestations, including urticarial-like lesions, generalized erythema and papules, plaques and nodules, leukocytoclastic vasculitis, erythema multiforme, indurated erythematous lesions, oral ulcerations, alopecia, and malar erythema [2, 5, 9, 11-12]. Furthermore, the cutaneous manifestations of KFD could be consistent with cutaneous manifestations of SLE [13].

Although laboratory findings are usually normal, abnormalities may include leukopenia, anemia, elevated ESR and C-reactive protein (CRP), lactate dehydrogenase (LDH), and aminotransferases [13-14]. Diagnosis is confirmed by lymph node biopsy with histopathological features, including paracortical foci with necrosis and a histiocytic cellular infiltrate [9-10, 12]. Around 15% of patients with KFD have been noted to have a weakly positive ANA with the manifestation of full-blown SLE [14].

KFD is a self-limiting condition in which symptoms usually resolve spontaneously within one to four months, recurring in only 3% - 4% of patients [15]. Treatment is usually supportive with analgesics, although corticosteroids, hydroxychloroquine, methotrexate, and intravenous immunoglobulins may be used in severe cases [5, 16-17].

Our presented case includes a unique combination of cutaneous, biochemical, and pathological findings; thus, a diagnostic challenge was posed in delineating the distinction between KFD and SLE, both clinically and pathologically [2]. Points in our case report that challenge the possibility of erosive lichen planus being a cutaneous manifestation of KFD include the chronic course of the heel ulcerations, as the typical course of KFD is acute to subacute, presence of confounding factors (SLE), and absence of similar reported case reports and supporting evidence in current literature.

## Conclusions

KFD poses significant diagnostic challenges to both clinicians and pathologists. As such, one should remain alert to the possibility of KFD, a known mimicker of serious systemic diseases, including lymphoma, when a young individual presents with a constellation of symptoms, including cutaneous involvement and lymphadenopathy. In addition, the following case report emphasizes the association between KFD and SLE and the less common clinical presentation of intra-abdominal lymphadenopathy in patients with KFD. Finally, whether erosive lichen planus is to be considered as a cutaneous manifestation of KFD still requires further supporting evidence in the literature.
